# Material Analysis and Molecular Dynamics Simulation for Cavitation Erosion and Corrosion Suppression in Water Hydraulic Valves

**DOI:** 10.3390/ma13020453

**Published:** 2020-01-17

**Authors:** Masoud Kamoleka Mlela, He Xu, Feng Sun, Haihang Wang, Gabriel Donald Madenge

**Affiliations:** 1 College of Mechanical and Electrical Engineering, Harbin Engineering University, Harbin 150001, China; masoudkamoleka@gmail.com (M.K.M.); sunfeng@hrbeu.edu.cn (F.S.); wanghaihang@hrbeu.edu.cn (H.W.); 2 College of Aerospace and Civil Engineering, Harbin Engineering University, Harbin 150001, China; gmadenge@icloud.com

**Keywords:** polymer, water hydraulic valve, cavitation, erosion, corrosion, coating selection, molecular dynamics

## Abstract

In the milestone of straggling to make water hydraulics more advantageous, the choice of coating polymer for water hydraulics valves plays an essential role in alleviating the impact of cavitation erosion and corrosion, and this is a critical task for designers. Fulfilling the appropriate selection, we conflicted properties that are vital for erosion and corrosion inhibitors, as well as the tribology in the sense of coefficient of friction. This article aimed to choose the best alternative polymer for coating on the selected substrate, that is, Cr_2_O_3_, Al_2_O_3_, T_i2_O_3_. By applying PROMETHEE (Preference Ranking Organization Method for Enrichment Evaluations), the best polymer obtained with an analyzed performance attribute is Polytetrafluoroethylene (PTFE) that comes up with higher outranking (0.5932052). A Molecular Dynamics (MD) simulation was conducted to identify the stronger bonding with the regards of the better cleave plane between Polytetrafluoroethylene (PTFE) and the selected substrate. Polytetrafluoroethylene (PTFE)/Al_2_O_3_ cleaved in (010) plane was observed to be the strongest bond in terms of binding energy (3188 kJ/mol) suitable for further studies.

## 1. Introduction

Today, water hydraulics still face some major adversity to expand their application. Generally, the initial cost of hydraulic fluid components is higher than the hydraulic oil components. This property can be reduced using a deficient cost pressure medium (water), much lower insurance, and disposal costs, if the time used is long enough [1]. Specifically, water hydraulics have a more rapid response and better efficiency compared to oil hydraulics. They are also more stable (in terms of flow velocity and efficiency) over a wide range of operating temperatures due to the higher volume module of water (about 50% higher than that of mineral oil). They also have a lower viscosity (less than 1/30 at 50 °C mineral oil) and higher specific heat capacity (multiple of 2.2 higher than mineral oil). All of the above benefits make water hydraulics particularly interesting for robotics for high-performance actuation techniques, in addition to improving people’s awareness of environmental protection and sustainable development requirements [2,3,4,5]. Hydraulic systems that utilize water as a pressure medium could be the correct solution for the environmental and safety problems of most oil hydraulic systems [6]. Water hydraulic systems have been widely used in the fields of steel and glass production, ocean exploration, food and medicine processing, and coal mining [3,7]. Water-based systems are highly profoundly vulnerable to cavitation. The imploding of vapour cavities inside the flow motivates pressure pulsations, which may conjointly cause intense noise, part vibration, energy loss, erosion and corrosion of internal surfaces and eventually, scale back the performance of the system or failure of the part [8,9]. In rivers and seawater, several works studied the mechanism of cavitation, erosion abrasion, and corrosion. Cavitation bubbles are created when the fluid’s static pressure drops below the vapour pressure at a specific temperature. [3,8]. The formation and collapse of bubbles of vapor would result in high local temperatures and high pressure [10]. The valve is one of the critical hydraulic components and due to the sharp drop in pressure caused by throttling, the occurrence of cavitation is normal [10]. Significantly, fluid valves can be classified as non-continuous valves (e.g., shut-off and change valves) and continuous valves (e.g., servo and proportional valves) [11]. There are already numerous water hydraulic power control systems on the market. Usually, their components are made of stainless steel to ensure satisfactory performance in harsh, conventional operating conditions. They do not, however, provide the necessary quality and long-term, low-friction, and low-wear efficiency [6]. Tensile properties are made up of the materials’ reaction to resist when tension forces are applied. It is vital to establish tensile properties because it provides information on the elasticity module, elastic limit, elongation, proportional limit, area reduction, tensile strength, yield point, yield strength, and other tensile properties [12]. Fairfield in [13] declares that the studied erosion resistance was a substitute for a primarily unknown combination of other properties, including fracture resistance, strength, impact resistance, surface roughness, hardness, and service temperature limitation. He additionally clarifies that material properties with the largest contribution to jets resistance are compressive, tensile, and flexural, without forgetting surface roughness, thickness, peak service temperature, and thermal conductivity. Due to polymer significance in many applications, the characterization and study of superhydrophobic surfaces are of significant interest. Many material science studies have focused on surfaces with thrilling behaviors of wettability, such as superhydrophobic and superhydrophilic. The surface must have hydrophobic chemistry for the coating of polymer with other metal oxides of choice. The angle of contact is the angle that a liquid and solid surface create when they come into contact. This angle depends on both the material properties and the attractive and repulsive interaction between the materials. When the liquid spreads over the surface, it forms a small contact angle, whereas the contact angles are higher when the contact area between the surface and the liquid is smaller. The surface properties were analyzed and showed a good anti-corrosion ability [14]. The estimation of polymer solubility in solvents is one of the most important applications of solubility parameters [15]. Joel H. Hildebrand in [16] (who performed definitive work on the solubility of non-electrolytes in 1916 and lay the groundwork for solubility theory) recommended the square root of the cohesive energy density as a numerical value showing a particular solvent’s solvency behavior [15]. The cohesive energy density is a numerical value describing the vaporizing energy in calories per cubic centimeter and a direct representation of the severity of van der Waals forces binding the substance’s well-organized molecules [17]. Nevertheless, water’s low viscosity, strong corrosion, and inadequate lubricating capacity have created significant challenges for this component’s development and application. It is, therefore, an urgent task to screen materials suitable for the hydraulic water valve, which is the purpose of this research [18]. Hardness has long been considered a key criterion for the measurement of wear resistance. The erosion rate is correlated with these material properties, such as dynamic hardness, critical failure strain, and toughness [19].

## 2. Materials and Methods

### 2.1. Polymer Selection for Water Hydraulics Valves

In the engineering design and manufacturing process, the material plays a vital role. The appropriate selection of materials for a specific function is one of the designers’ vital tasks. To meet the end specifications of the consumer, developers need to evaluate, with specific functionalities, the quality of various materials and find appropriate materials. The selection of materials is a challenging and time-consuming task because of the presence of a large number of materials with different attributes. [20]. The advancement of MCDA methods was not only inspired by a variety of real-life issues that need to address several considerations but also by experts’ ability to incorporate stronger decision-making methods using recent developments in mathematical modeling, statistical analysis, and computer technology. To date, many computational approaches have been developed and applied to solve problems of material selection arising from various fields of engineering [20]. The PROMETHEE (Preference Ranking Organization Method for Enrichment Evaluations) approach is one of the latest MCDA methods developed by Brans [21] and further extended by Vincke and Brans [22].

**Figure 1:**
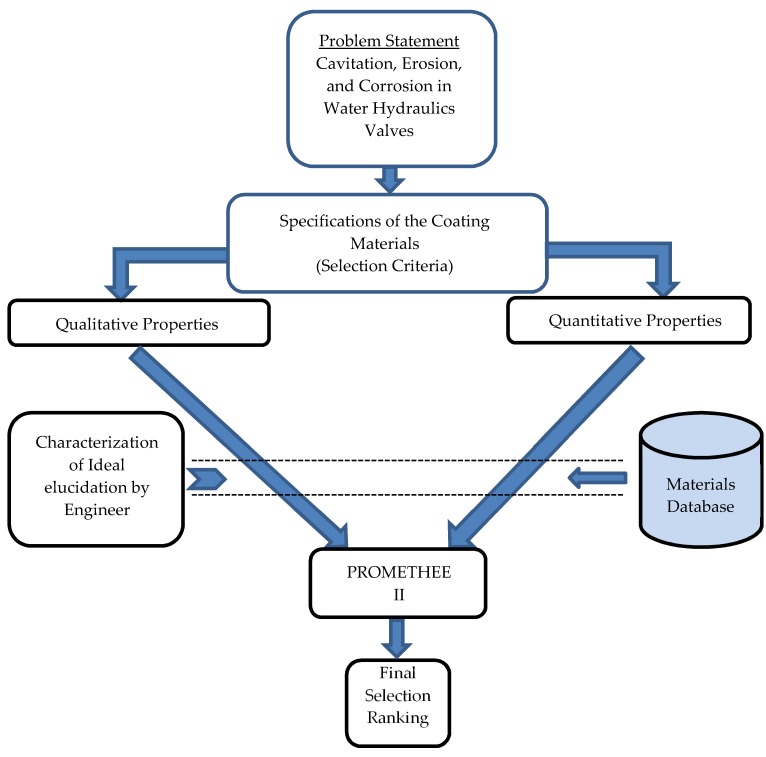
Flowchart for polymer coating selection.

PROMETHEE is an outranking method for a finite set of alternative actions to be ranked and selected among the often widely divergent criteria. PROMETHEE is, indeed, a straightforward design and application ranking method compared to other multi-criteria analysis methods [23]. This part will deal with the polymer coating selection and particularly utilizing the PROMETHEE (Preference Ranking Organization Method for Enrichment Evaluations) method. Figure 1 shows a flowchart for coating selection that uses the PROMETHEE technique, and Table 1 shows the used criteria used for polymer selection.

#### 2.1.1. Polyvinylchloride (PVC)

**Figure 2:**
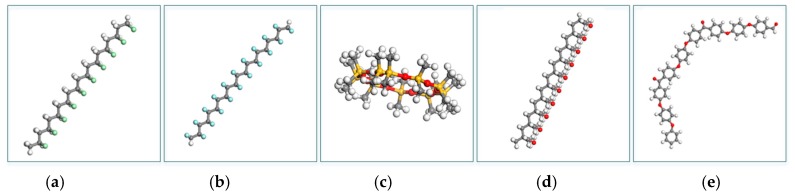
Shows (**a**) Polyvinylchloride; (**b**) Polytetrafluoroethylene; (**c**) Polydimethylsiloxane; (**d**) Polymethylmethacrylate; (**e**) Polyaryletheretherketone.

PVC was examined as amongst the most chemically antagonistic polymers and hydrophilicity [24]. Generally, PVC as seen in Figure 2a dissolves into polar solvents but is very resistant to hydrocarbons, alcohols, esters, acids, bases, and salts. Its chemical defiance can be predicted using solubility parameters [25]. When warmed exceedingly its T_g_ (about 87 °C), unstabilized PVC encounters dehydrochlorination [26]. PVC is the third most widely produced polymer in the world, with its outstanding mechanical strength, flame choke off, thermal stability, and insulation peculiarity, and has been mostly used as a contentedness resin in many products [27]. Table 1 gives some useful PVC properties for water hydraulics valves.

#### 2.1.2. Polytetrafluoroethylene (PTFE)

Polytetrafluoroethylene (PTFE) in Figure 2b also recognized as Teflon. This material notably used in spacecraft design, automotive industries, and semiconductor design [38]. Disclosed utilization of the PTFE is divulging with the fact that it has meritorious chemical inactivity and high thermal stability. The coefficient of sliding friction between PTFE and many engineering materials is extremely low and has the self-lubrication capability [39,40]. It is insoluble in all common solvents and is resistant to almost all acidic and caustic substances [40]. PTFE is resistant to attack even by corrosive solutions and is practically unaffected by water (hydrophobicity) [26]. PTFE is subjected and considered as superplastic. Table 1 provides some useful PTFE properties suitable for water hydraulic valves. The essential characteristic of a superplastic material is its high strain rate sensitivity of flow stress that entertains high resistance [41]. Although PTFE has a high impact strength, its tensile strength and resistance to wear and creep are low compared to other polymers in the materials’ database. Polytetrafluoroethylene is primarily used in applications needing extreme strength, exceptional chemical and heat tolerance, good electrical properties, low friction, or a blend of these properties [26].

#### 2.1.3. Polydimethylsiloxane (PDMS)

The Figure 2c shows a silicon-based elastomer Polydimethylsiloxane which has several interesting properties, along with biological and chemical inertness, extremely low T_g_, high temperature, and oxidation ability to resist, and vapor permeability [42]. After preparing the PDMS solid, the hydrophilic uniqueness of the PDMS was lowered so that it can be covered with metals for electrode and microchannel applications [43]. Table 1 provides some vital water hydraulic valve PDMS properties.

#### 2.1.4. Polymethylmethacrylate (PMMA)

Poly (methyl methacrylate) as shown in Figure 2d, is the most significant member of acrylic polymers, distinguished by its thrilling weatherability, strong crystallinity, and extreme glassiness to visible light. It is mechanically robust with excellent insulating properties, making this thermoplastic polymer a choice for many mechanical, microelectronic and electrical uses, and good chemical and electrical and thermal tolerance [26,44]. In the arena of mechanical strength, PMMA has a low elongation at breakage and a high Young’s Modulus. Therefore, it does not splinter upon rupture and happens to be one of the hardest thermoplastics with high scratch resistance [45]. Table 1 presents some useful PMMA properties for valves for water hydraulics.

#### 2.1.5. Polyaryletheretherketone (PEEK)

Polyetheretherketone (PEEK) is a tough semi-crystalline thermoplastic polymer with excellent mechanical and dielectric properties, as well as good chemical resistance [39,46]. Polyetheretherketone (PEEK) as seen in Figure 2e is a member of the Polyaryletherketone group that exhibit a strong-performance polymer (HPP) that is commonly used in relatively movement applications. It has extreme chemical resistance; it is suitable for tribological applications due to its high strength and wears resistance. Polyaryletheretherketone (PEEK) has outstanding properties due to its semi-crystalline structure and the molecular strength of its repeating units [46].

### 2.2. PROMETHEE (Preference Ranking Organization Method for Enrichment Evaluations) II

Material selection for engineering design is a multicriteria decision-making model (MCDM) problem that requires consideration of several available materials and conflicting evaluation criteria. The materials screened to be under selection are designated as P1 (Polyvinylchloride), P2 (Polytetrafluoroethylene), P3 (Polydimethylsiloxane), P4 (Polymethylmethacrylate) and P5 (Polyaryletheretherketone), as seen in Table 2. In addition, the beneficial and non-beneficial attributes or criteria to perform well in the water-logged condition of water hydraulic valves under the impact of water cavitation were chosen.

**Figure 3:**
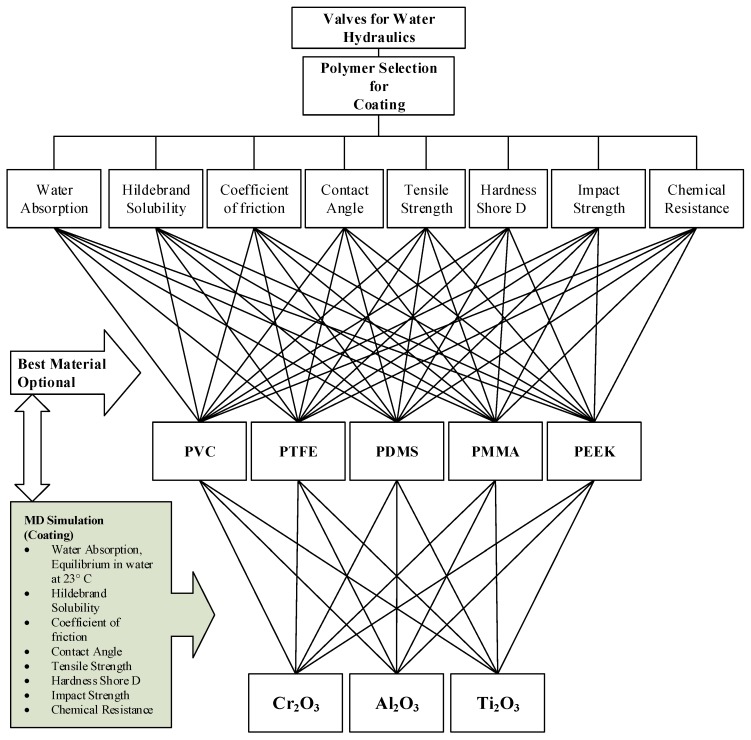
The hierarchy of the Material Selection.

Figure 3 shows the hierarchy of the Material Selection based on the non-beneficial and beneficial criteria. Among the non-beneficial criteria are water absorption or equilibrium in the water at 23 °C (A1), Hildebrand Solubility (A2), and Coefficient of friction (A3). The beneficial criteria are Contact angle (A4), Tensile strength (A5), Hardness Shore D (A6), Impact Strength (A7) and Chemical resistance (A8). The criterion must fit the requirement of the system to survive from the prone to hydrodynamic cavitation surge pressure and erosion wears due to massive pressurized fluid flow in the valve chamber. Corrosion of the valve’s internal wall must be prevented to sustain the longer life of the valves and to resist the erosion notch. Water absorption or equilibrium in the water at 23 °C is based on the superhydrophobic surface. However, as a result, the low viscosity and poor lubrication properties of water lead to potential risks of higher wear and friction, especially in the proportional spool valves [6].

**Figure 4:**
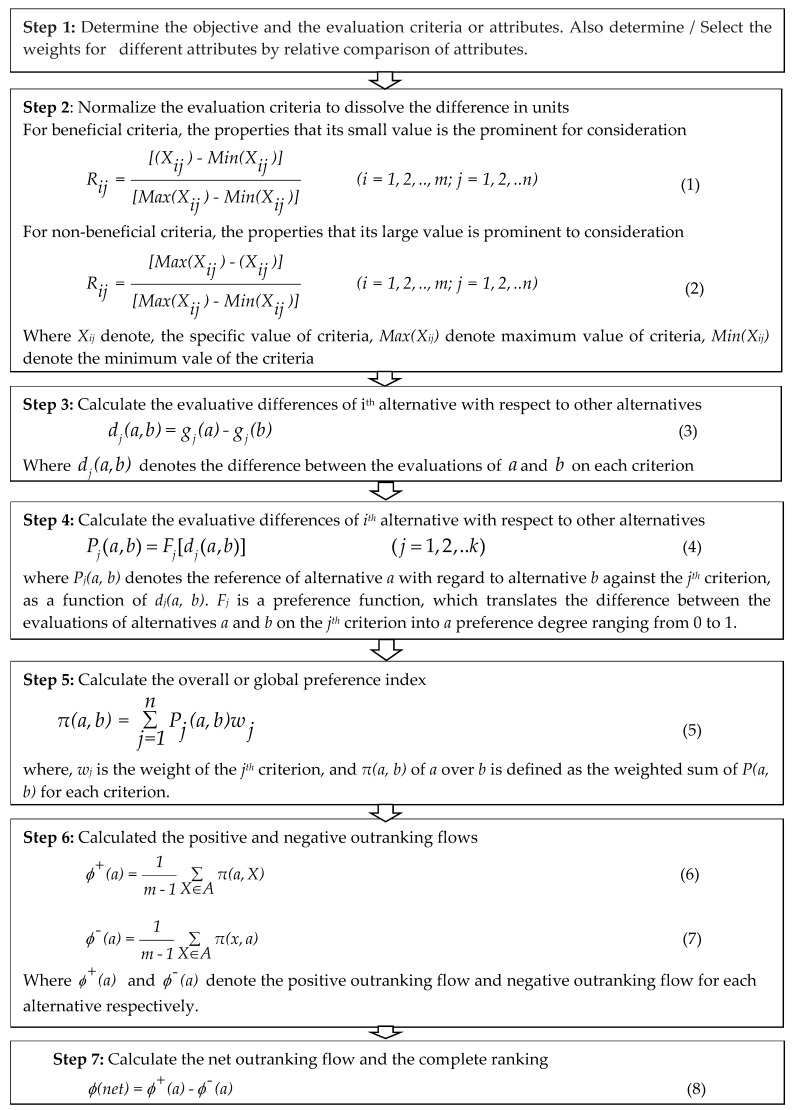
Stepwise complete ranking PROMETHEE II.

For some materials, hardness (macro and micro) is a good cavitation erosion resistance indicator [47]. In terms of hydrophobicity, water absorption is commonly inferred as a measure of a solute’s relative tendency to prefer a non-aqueous environment rather than an aqueous one. Hydrophobicity plays an essential role in the biological and physicochemical behaviour of numerous types of organic compounds [48]. Simply, hydrophobicity composite material, remarkable mechanochemical robustness, stain repellency, oil-water separation. Figure 4 presents a stepwise procedure for implementing PROMETHEE II [49,50]. Based on the requirement of the valve for water hydraulics and considering the flow of most significant selected attributes, engineering and material fields are integrated by aggregating the significant weights of criteria and the ratings of alternatives. Table 6 applies the weight distribution of the selected attribute. 

### 2.3. Molecular Dynamics Simulation to Predict the Substrate and Suitable Coating Plane

**Figure 5:**
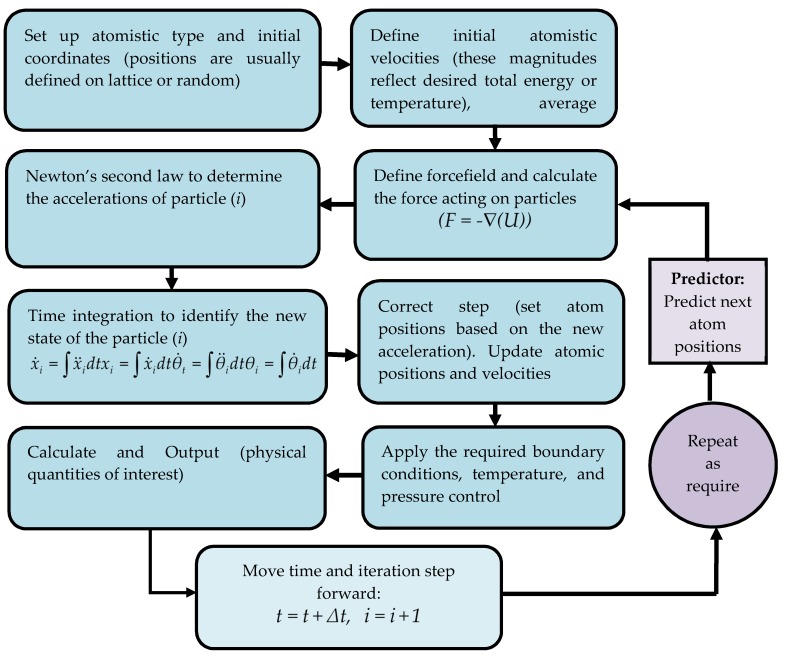
Molecular dynamics simulation algorithm.

MD simulation is a modeling technique based on physics that has been commonly used to represent different material systems as it can provide detailed information on variations and conformational changes in the structure and behavior of material systems at the molecular level [51,52]. Figure 5 below shows the Molecular dynamics algorithms. Here, the MD Simulation was conducted to predict a polymeric coating on the selected substrates (Cr_2_O_3_, Al_2_O_3_, and Ti_2_O_3_) along the planes (100), (010) and (001). The simulation computation was created using the material science simulation software developed by Accelrys Software Inc. in San Diego, CA, USA, named Material Studio ^®^ Software, after several phases of model design and calculation. The tasks were to create engineered Cr_2_O_3_, Al_2_O_3_, and Ti_2_O_3_, slabs with an equal thickness of 13.233 × 10^−10^ m each in a cleaved plane along (100), (010) and (001) each. Using a smarter algorithm with a maximum iteration of 100,000 steps, the surfaces were relaxed at minimum energy using model typing for force-field and condensed-phase optimized molecular potentials (COMPASS) to ensure bonding between metal oxide (Cr_2_O_3_, Al_2_O_3_, and Ti_2_O_3_). The supercell was designed by growing the surface to the lattice vector of U × V = 6 × 6 and then constructing the slab without the vacuum in order to change the periodicity from 2D to 3D. The steps of making a slab are the same as all-metal oxides (Cr_2_O_3_, Al_2_O_3_, and Ti_2_O_3_) picked. The periodic layer of the amorphous polytetrafluoroethylene cell (PTFE) was constructed and the structure was geometrically refined from the atactic polymer used in this work. This was pre-constructed, manufactured from the 50-chain length repeat-unit tetrafluoroethylene with a number of configurations set to 1.0, the final design goal density agreed to 2 g/cm^3^. The next step was to build a layer with a vacuum of 70 × 10^−10^ m. Minimized polytetrafluoroethylene was deposited on the matched metal oxide slabs of Cr_2_O_3_, Al_2_O_3_, and Ti_2_O_3_.

A molecular dynamics simulation was conducted in each coating composite layer. The temperature control method of Andersen was used, ensembles of a constant number of particles, constant volume, and constant temperature (NVPT). Each arrangement of molecular dynamics (MD) simulation was done with an interval of 1 femtosecond (fs) and each frame of 5000. COMPASS forcefield was determined in all the arrangements. Simulation steps were set to 200,000 with a total dynamic time of 200 picoseconds (ps).

## 3. Results and Discussion

**Figure 6:**
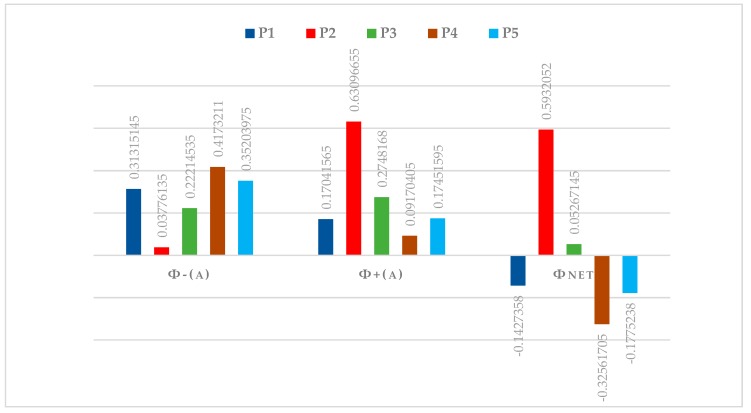
PROMETHEE I-II partial and complete ranking.

**Figure 7:**
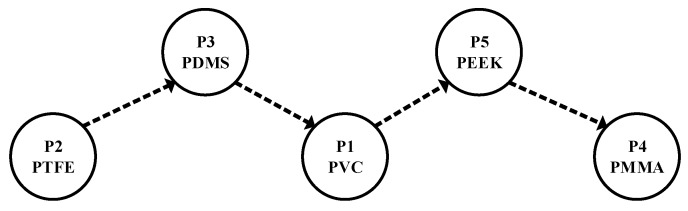
Ranking sequence.

PROMETHEE was used so solve the polymer selection problem based on the Equations (1)–(8) as shown in the Table 3, Table 4, Table 5, Table 6, Table 7 and Table 8. The PROMETHEE analysis was done by following all the essential steps of the analysis. Finally, the PTFE was the best polymer based on the selected significant properties for coating in the valve in water hydraulics. Table 9 shows the net flow of polymer alternatives. The PTFE has a higher value of 0.5932052, as seen in Figure 6 of the PROMETHEE I-II partial and complete ranking that the (*Φnet* values) of alternative P2 is the better coating polymer for water hydraulics valves to resist the cavitation erosion and corrosion. Moreover, PDMS, PVC, PEEK, and PMMA, as shown in Figure 7 of the ranking sequence, follow PTFE.

Water hydraulics valves are subjected to cavitation erosion, wear, and corrosion due to a high bubbles implosion impact; selected properties of PTFE fit well with the coating requirements. Furthermore, the molecular dynamics results show that the PTFE coated with Al_2_O_3_ in the cleaved plane of (010) gives more considerable binding energy compared to other composites in all the cleaved planes. This may be due to difference in the chemical reactivity functions within the frameworks of the density functional theory (DFT), the high rate of transfer of electrons to or from the reactants in the plane (010) of PTFE/Al_2_O_3_, or due to the change in the geometric structure of the molecules (equivalent in external potential). In addition, the chemisorption takes place due to the strong electrostatic (ionic) bond.

**Figure 8:**
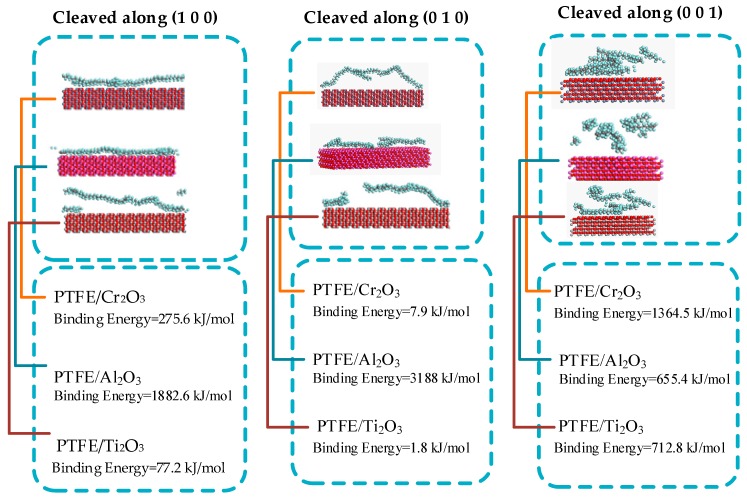
Molecular dynamics Polytetrafluoroethylene coating on Metal Oxides (Ti_2_O_3_, Al_2_O_3_, and Cr_2_O_3_).

These results show the sign of promising the strength of the composite over the effect of the impairment of water hydraulics valves due to the sudden surge pressure caused by the imploding of cavitation bubbles. Figure 8 below shows the pictorial presentation and data obtained after simulation.

## 4. Conclusions

The selection and screening of polymer coating on metal oxides were studied for the design of liquid hydraulic valves. For this reason, PROMETHEE’s decision-making methods were used. The results show the most suitable polymer materials being simulated. The improvement in the cavitation erosion resistance can mainly be linked to the increase in hardness and the elastic response of the PTFE/Al_2_O_3_ enhancement. Allison and Tong, in [53], using the density functional theory, depicted that binding energy is proportional to hardness. Moreover, simulation of molecular dynamics found the best substrate and cleaved plane in further studies should be considered by the characterization of a different PTFE/Al_2_O_3_ composition ratio.

## Figures and Tables

**Table 1:** Selected polymers and properties that are suitable for water hydraulic valves.

	Attribute or Criteria	Units	Polymer					Reference
			P1	P2	P3	P4	P5	
Non-beneficial	A1	%	0.4	0	0.17	0.3	0.5	[28,29]
	A2	(MPa^0.5^)	19.1	12.7	14.9	21.3	22.8	[28]
	A3	-	0.8	0.06	0.8	0.539	0.4	[28,30,31]
Beneficial	A4	(degree)	91.9	122	110	74.7	90	[28]
	A5	MPa	51.7	35	9.7	72.4	103	[32,33,34]
	A6	D	25	50	70	78	88	[28,35,36]
	A7	J/m	200	188	22	20	80	[17,28,37]
	A8	-	Satisfactory	Excellent	Good	Poor	Very Good	[28]

Annotation: P1 is the polyvinylchloride, P2 is the polytetrafluoroethylene, P3 is the polydimethylsiloxane, P4 is the Polymethylmethacrylate, and P5 is the Polyaryletheretherketone. While non-beneficial criteria are water absorption or equilibrium in the water at 23° C (A1), Hildebrand Solubility(A2), and coefficient of friction(A3). The beneficial criteria are Contact angle (A4), Tensile strength (A5), Hardness Shore D (A6), Impact Strength (A7) and Chemical resistance (A8) [24,25,26,27].

**Table 2:** Polymer properties and introducing the 5-point scale.

Polymer	Attributes							
	A1	A2	A3	A4	A5	A6	A7	A8
**P1**	0.4	19.1	0.4	91.9	51.7	25	200	2
**P2**	0	12.7	0.06	122	35	50	188	5
**P3**	0.17	14.9	0.8	110	9.7	70	22	3
**P4**	0.3	21.3	0.539	74.7	72.4	78	20	1
**P5**	0.5	22.8	0.4	90	103	88	80	4

Annotation: Apply the 5-point scale for Chemical Resistance attribute (A8) in Table 1. Poor = 1, Satisfactory = 2, Good = 3, Very Good = 4 and Excellent = 5.

**Table 3:** Applying normalization to the evaluation criteria to obtain R_ij_ (beneficial and non-beneficial) using Equations (1) and (2).

Polymer	Attributes							
	A1	A2	A3	A4	A5	A6	A7	A8
P1	0.2	0.46835	0	0.36364	0.45016	0	1	0.25
P2	1	1.2848	1	1	0.37513	0.39683	0.93333	1
P3	0.66	1	0	0.7463	0	0.71429	0.01111	0.5
P4	0.4	0.18987	0.3527	0	0.67202	0.84127	0	0
P5	0	0	0.54054	0.32347	1	1	0.33333	0.75
Max (X_ij_)	0.5	22.8	0.8	122	103	88	200	5
Min (X_ij_)	0	14.9	0.06	74.7	9.7	25	20	1

**Table 4:** Applying the calculation of the evaluative differences of ith alternative with respect to other alternatives using Equation (3).

d_j_ (a,b)	Attributes							
	A1	A2	A3	A4	A5	A6	A7	A8
d(P1-P2)	−0.80	−0.81645	−1	−0.63636	0.07503	−0.39683	0.06667	−0.75
d(P1-P3)	−0.46	−0.53165	0	−0.38266	0.45016	−0.71429	0.98889	−0.25
d(P1-P4)	−0.2	0.27848	−0.3527	0.36364	−0.22186	−0.84127	1	0.25
d(P1-P5)	0.2	0.46835	−0.54054	0.04017	−0.54984	-1	0.66667	−0.5
d(P2-P1)	0.8	0.81645	1	0.63636	−0.07503	0.39683	−0.06667	0.75
d(P2-P3)	0.34	0.2848	1	0.2537	0.37513	−0.31746	0.92222	0.5
d(P2-P4)	0.6	1.09493	0.6473	1	−0.29689	−0.44444	0.93333	1
d(P2-P5)	1	1.2848	0.45946	0.67653	−0.62487	−0.60317	0.6	0.25
d(P3-P1)	0.46	0.53165	0	0.38266	−0.45016	0.71429	−0.98889	0.25
d(P3-P2)	−0.34	−0.2848	−1	−0.2537	−0.37513	0.31746	−0.92222	−0.5
d(P3-P4)	0.26	0.81013	−0.3527	0.7463	−0.67202	−0.12698	0.01111	0.5
d(P3-P5)	0.66	1	−0.54054	0.42283	−1	−0.28571	−0.32222	−0.25
d(P4-P1)	0.2	−0.27848	0.3527	−0.36364	0.22186	0.84127	−1	−0.25
d(P4-P2)	−0.6	−1.09493	−0.6473	−1	0.29689	0.44444	−0.93333	−1
d(P4-P3)	−0.26	−0.81013	0.3527	−0.7463	0.67202	0.12698	−0.01111	−0.5
d(P4-P5)	0.4	0.18987	−0.18784	−0.32347	−0.32798	−0.15873	−0.33333	−0.75
d(P5-P1)	−0.2	−0.46835	0.54054	−0.04017	0.54984	1	−0.66667	0.5
d(P5-P2)	−1	−1.2848	−0.45946	−0.67653	0.62487	0.60317	−0.6	−0.25
d(P5-P3)	−0.66	−1	0.54054	−0.42283	1	0.28571	0.32222	0.25
d(P5-P4)	−0.4	−0.18987	0.18784	0.32347	0.32798	0.15873	0.33333	0.75

**Table 5:** Application of the preference function using Equation (4).

P_j_(a,b)	Attributes							
	A1	A2	A3	A4	A5	A6	A7	A8
d(P1-P2)	0	0	0	0	0.0750	0	0.0667	0
d(P1-P3)	0	0	0	0	0.4502	0	0.9889	0
d(P1-P4)	0	0.2785	0	0.3636	0	0	1	0.25
d(P1-P5)	0.2	0.4684	0	0.0402	0	0	0.6667	0
d(P2-P1)	0.8	0.8165	1	0.6364	0	0.3968	0	0.75
d(P2-P3)	0.34	0.2848	1	0.2537	0.3751	0	0.9222	0.5
d(P2-P4)	0.6	1.0949	0.6473	1	0	0	0.9333	1
d(P2-P5)	1	1.2848	0.4595	0.6765	0	0	0.6	0.25
d(P3-P1)	0.46	0.5317	0	0.3827	0	0.7143	0	0.25
d(P3-P2)	0	0	0	0	0	0.3175	0	0
d(P3-P4)	0.26	0.8101	0	0.7463	0	0	0.0111	0.5
d(P3-P5)	0.66	1	0	0.4228	0	0	0	0
d(P4-P1)	0.2	0	0.3527	0	0.2219	0.8413	0	0
d(P4-P2)	0	0	0	0	0.2969	0.4444	0	0
d(P4-P3)	0	0	0.3527	0	0.6720	0.1270	0	0
d(P4-P5)	0.4	0.1899	0	0	0	0	0	0
d(P5-P1)	0	0	0.5405	0	0.5498	1	0	0.5
d(P5-P2)	0	0	0	0	0.6249	0.6032	0	0
d(P5-P3)	0	0	0.5405	0	1	0.2857	0.3222	0.25
d(P5-P4)	0	0	0.1878	0.3235	0.3280	0.1587	0.3333	0.75

**Table 6:** Applying the calculation of the overall or global preference index, using Equation (5).

	Attributes								*π*(*a*,*b*)
Weight →	A1	A2	A3	A4	A5	A6	A7	A8	
	0.2	0.18	0.08	0.16	0.06	0.06	0.14	0.12	
w_j_ × d(P1-P2)	0	0	0	0	0.0045	0	0.0093	0	0.0138356
w_j_ × d(P1-P3)	0	0	0	0	0.0270	0	0.1384	0	0.1654542
w_j_ × d(P1-P4)	0	0.0501	0	0.0582	0	0	0.14	0.03	0.2783088
w_j_ × d(P1-P5)	0.04	0.0843	0	0.0064	0	0	0.0933	0	0.224064
w_j_ × d(P2-P1)	0.16	0.1470	0.08	0.1018	0	0.0238	0	0.09	0.6025884
w_j_ × d(P2-P3)	0.068	0.0512	0.08	0.0406	0.0225	0	0.1291	0.06	0.4514746
w_j_ × d(P2-P4)	0.12	0.1971	0.0518	0.16	0	0	0.1307	0.12	0.7795376
w_j_ × d(P2-P5)	0.2	0.2313	0.0368	0.1082	0	0	0.084	0.03	0.6902656
w_j_ × d(P3-P1)	0.092	0.0957	0	0.0612	0	0.0429	0	0.03	0.32178
w_j_ × d(P3-P2)	0	0	0	0	0	0.0191	0	0	0.0190476
w_j_ × d(P3-P4)	0.052	0.1458	0	0.1194	0	0	0.0016	0.06	0.3787868
w_j_ × d(P3-P5)	0.132	0.18	0	0.0677	0	0	0	0	0.3796528
w_j_ × d(P4-P1)	0.04	0	0.0282	0	0.0133	0.0505	0	0	0.1320038
w_j_ × d(P4-P2)	0	0	0	0	0.0178	0.0267	0	0	0.0444798
w_j_ × d(P4-P3)	0	0	0.0282	0	0.0403	0.0076	0	0	0.076156
w_j_ × d(P4-P5)	0.08	0.0342	0	0	0	0	0	0	0.1141766
w_j_ × d(P5-P1)	0	0	0.0432	0	0.0330	0.06	0	0.06	0.1962336
w_j_ × d(P5-P2)	0	0	0	0	0.0375	0.0362	0	0	0.0736824
w_j_ × d(P5-P3)	0	0	0.0432	0	0.06	0.0171	0.0451	0.03	0.1954966
w_j_ × d(P5-P4)	0	0	0.0150	0.0518	0.0197	0.0095	0.0467	0.09	0.2326512

**Table 7:** Applying the calculation of the positive and negative outranking flows, using Equations (6) and (7).

Aggregate Preference Function	P1	P2	P3	P4	P5	Leaving Flow *ϕ^+^(a)*
P1	-	0.0138356	0.1654542	0.2783088	0.224064	0.17041565
P2	0.6025884	-	0.4514746	0.7795376	0.6902656	0.63096655
P3	0.32178	0.0190476	-	0.3787868	0.3796528	0.2748168
P4	0.1320038	0.0444798	0.076156	-	0.1141766	0.09170405
P5	0.1962336	0.0736824	0.1954966	0.2326512	-	0.17451595
**Entering Flow *ϕ^−^(a)***	0.31315145	0.03776135	0.22214535	0.4173211	0.35203975	

**Table 8:** Applying the Equation (8) to calculate the net outranking flow and the complete ranking.

Polymer	Leaving Flow	Entering Flow	Net Flow	Ranking
P1	0.17041565	0.31315145	−0.1427358	3
P2	0.63096655	0.03776135	0.5932052	1
P3	0.2748168	0.22214535	0.05267145	2
P4	0.09170405	0.4173211	−0.32561705	5
P5	0.17451595	0.35203975	−0.1775238	4

**Table 9:** The value of the net flow of polymer alternatives.

Alternative Polymer	P1	P2	P3	P4	P5
*ϕ_net_*	−0.1427358	0.5932052	0.05267145	−0.32561705	−0.1775238
